# Editors’ Book Table

**Published:** 1875-08

**Authors:** 


### Editors' Book Table.

[NOTE. — All works reviewed in the pages of the CHICAGO MEDICAL JOURNAL may be found in the extensive stock of W. B. KEEN, COOKE & Co., whose catalogue of Medical Books will be sent to any address upon request.]

ANNUAL ADDRESS before the Society of the Alumni of the Medical Department of the University of Pennsylvania. By *Cornelius G. Comegys, M.D.*, Lecturer on Clinical Medicine in the Cincinnati Hospital, Ohio, etc., etc.

The above address is worthy of notice inasmuch as it is above the average of similar productions, and moreover presents some noteworthy propositions. Commencing with an eloquent tribute to the old Professors of the Faculty of Medicine of the University, both living and dead, the author proceeds to the consideration of "the position and responsibility of medical men." Under this head, he urges upon medical men the necessity of devoting more time and attention to the subject of "State Medicine," *i. e.*, public hygiene, and to a more active "participation in legislation," with the end to direct and control it in this relation. Not only does he advocate the exercise of the influence of the profession in the political arena, to the end that their knowledge might be utilized for the protection of public health, but likewise for the more judicious selection of men for positions of public trust and responsibility, who should be, by reason of more perfect mental and moral organization, better fitted for the discharge of important trusts, requiring both intelligence and integrity for their satisfactory fulfillment.

The author's opinions of the relations of mind and matter, are embodied in the expression—"This supreme mental force—will—is no transcendental entity to be considered apart from physical existence, but may be said, in its fullness, to be the correlative of the totality of the organic power of the brain." Hence, he urges the necessity for the exercise of educated judgment in the selection of leaders. His defense of the profession against the charges of superficiality and uncertainty of knowledge is able and just, and his advocacy of the claims of medicine upon private and public benevolence, well founded and forcible. He asserts truthfully, that "the resources of medical education have been derived from the profession itself." "Millions of dollars have been given to other colleges, but no one has constructed a medical edifice, or so endowed a medical school that its teachers should not be dependent on students' fees. Nevertheless, under all the embarrassing circumstances, our faculties have provided buildings, accumulated museums, libraries, and apparatus, at a vast expense, and have moreover assumed the gratuitous care of all the sick poor of hospitals and dispensaries. It is an unparalleled spectacle, and nowhere seen in the world but in the United States of America." The author presents his views upon the momentous question of medical education, which are worthy of study. Indeed, the whole address is evidently the production of a profound student, a liberal thinker, and an accomplished physician. H.

CERTAIN NERVOUS AFFECTIONS OF THE THROAT. By *Clinton Wagner*, *M.D.*, Physician to the Metropolitan Throat Hospital ; late Clinical Assistant, Hospital for Diseases of the Throat. London, etc., etc.

An interesting abstract, read before the New York Neurological Society, of some of the work of the Metropolitan Throat Hospital, organized Jan. 3, 1874, under the special charge of Drs. Clinton Wagner and J. Morris Asch, both late of the U. S. Army, with an efficient staff of consulting surgeons and clinical assistants.

This institution is the first systematized effort to specialize the study of laryngoly, in the United States, and thus far, as exhibited in the pamphlet before us, has been eminently successful. H.

A PRACTICAL TREATISE ON THE MEDICAL AND SURGICAL USES OF ELECTRICITY, including Localized and General Faradization; Localized and Central Galvanization; Electrolysis and Galvano-Cautery. By *Geo. M. Beard*, *A. M.*, *M.D.*, Member of the New York Society of Neurology and Electrology, etc., and *A. D. Rockwell*, *A.M.*, *M.D.*, Member of the New York Society of Neurology and Electrology, etc., etc.

The work of Drs. Beard and Rockwell became so widely and favorably known in its first edition, that but little is left to be said of this, the second, further than that it bears the impress of careful revision of the former, and presents moreover some valuable additional matter, especially in the department of electro-physics, and in that of electro-physiology and clinical electro-medicine likewise.

The authors' remarks on the necessity resting upon every one pretending to the use of electro-therapeusis to become first an electrologist, are worthy of careful attention. It is certain that had the therapeutic application of this force been restricted to "masters of electricity in its physical and physiological" relations, the range of its applicability would have been much more clearly defined, and its results more satisfactory.

The work is one of the most complete summaries of the whole science of electrology extant, and should be carefully studied by every one desiring to become expert therein.

The mechanical and typographical excellence of the book is assured by the imprimatur of William Wood & Co., New York. 1875. H.

A Practical Treatise on Eczema, including its Lichenous and Impetiginous Forms. By *Dr. McCall Anderson*, Professor of Clinical Medicine in the University of Glasgow; Physician to the Royal Infirmary, to the Dispensary for Skin Diseases, and to the Cutaneous Wards of the Western Infirmary, etc., Glasgow. Third Edition, with Illustrations. Philadelphia: Lindsay & Blakiston. 1875.

One of the most satisfactory books which has issued from the press during the current year, which no one, we think, can read without deriving both pleasure and profit therefrom. And no one, who has had even a few cases of the troublesome and obstinate malady in his care, will feel that the time spent in the study of this work has been lost.

The author's definition of eczema is clear and concise, and his description graphic. The etiology, pathology and therapeusis of the disease as it has occurred under his observation, are elaborated with much care and with a detail which, while circumstantial is never tedious. He has, moreover, collated his own observations, for making which, his opportunities have been singularly ample, with those of contemporary writers, thus presenting a copious array of corroborative testimony. His remarks upon the neurotic origin of eczema might have been elaborated a little more fully with advantage, for while he refers to it as a "transferred neurosis," with illustrated cases, he nowhere indicates its existence —which is of frequent occurrence—as a reflex neurosis from remote peripheral irritation.

The mechanical execution, including paper, presswork and type, is really beautiful—the arrangement of marginal titles greatly facilitating study. We should be glad to recognize in this the beginning of another permanent improvement in the art of medical book making, which has in the hands of our more prominent publishers already made such rapid advances. H.

Orthopædia, or a Practical Treatise on the Aberrations of the Human Form. By *James Knight, M.D.*, Member of the Medico-Chirurgical Faculty of Maryland, the District Medical Society of Ohio, and the County Medical Society of New York; Physician and Surgeon in Charge of the Hospital of the New York Society for the Relief of the Ruptured and Crippled, etc., etc. New York: G. P. Putnam's Sons. 1874.

The practical results of thirty years of private and ten of hospital practice, in the special department of orthopædic surgery. The author has undoubtedly enjoyed immense advantages in accomplishing himself in his specialty, and appears to have utilized them fully, not only by observation, but by study of the literature of his subject, which he seems to have mastered from the earliest to its most recent contributions.

The book comprehends a very wide range of topics within the specialty, all of which are carefully considered. Some of the more remarkable cases detailed are illustrated by wood cuts, as are likewise the various forms of apparatus recommended. The work is thoroughly practical, and cannot fail to prove very useful. H.

On the Treatment of Pleurisy: With an Appendix of Cases, showing the value of Croton Oil, Ether, and Iodine, as Counter-irritants in other Diseases. By *John W. Corson, M.D.*, Late Physician to the Class of "Diseases of the Chest and Throat," in the New York and Eastern Dispensaries, etc., etc. New York: Wm. Wood & Co. 1874.

A pleasantly written narrative of the advantages to be derived from the use of mild measures generally, and especially of external remedies in the treatment of chest affections, as practically demonstrated in the large field of observation afforded in the New York Dispensary.

The little lecture of thirty pages will well repay the time spent in reading it. H.

A MANUAL OF DIET IN HEALTH AND DISEASE. By *Thomas King Chambers, M.D., Oxon., F.R.C.P.*, London, Honorary Physician to H. R. H. the Prince of Wales; Consulting Physician to St. Mary's and the Lock Hospitals; Lecturer on Medicine at St. Mary's School; Corresponding Fellow of the Academy of Medicine. New York, etc., etc. Philadelphia: Henry C. Lea. 1875.

To those familiar with the writings of Dr. Chambers, his claim to share with Sir Thomas Watson the title of Xenophon of Medical Literature, will be readily conceded, for it is certain that he has few equals and no superior, as a master of elegant English, within the ranks of professional writers. His well-earned reputation in this regard will suffer no diminution from the work before us.

The subject matter is arranged under three subdivisions, the first of which comprehends General Dietetics; the second, Special Dietetics of Health; and the third, Dietetics of Sickness. In the first is demonstrated, not only the essential constitution of a dietary system best adapted for the necessities of mankind in health, under varying conditions of existence, but likewise the great superiority, not only from a dietetic, but from an economic point of view, of a mixed diet, and one also which shall vary widely in its constituent elements, with the varying occupations and epochs of man's life. The second chapter, upon the choice of food, is not only instructive, but practically so, containing rules for the selection of the best meats, and their greater economic value. The sanitary relations of diseased meats and of animal parasites is condensed into a few comprehensive pages, so clearly and explicitly as to be intelligible to all. Dr. Chambers is an Englishman, writing in the smoky atmosphere of London, and hence we can account for his very feeble commendation of fruits as food. Had his lot been cast in sunnier lands where nature provides a more bountiful repast for her children, and where fruits constitute so large—sometimes the principal—portion of the food of all classes, his commendation would have been more liberal, his praise less stinted.

From the admirably written chapter upon wines as dietetics, it is easy to perceive that our author is no "crusader," for none of that illustrious army could write so *tastefully* of the delicious juice of the grape. What could be more refreshing on a day like this, with the mercury up in the nineties, than to hear from the lips of a wise physician like Dr. Chambers, that "as a regular beverage for a healthy person there is no wine in the English market equal to claret"? In this era of temperance run mad, this author is to be thanked by every honest lover of temperate festivity for these words: "But I am quite sure that the not infrequent manufacture of occasions for domestic rejoicing, a birthday, a wedding anniversary, a harvest home, a horse sold, the planting of a tree, the calving of a cow, a daughter presented at court, or cutting her first tooth, or any other good stroke of business, is a great promoter, not only of love and happiness, but of personal health. Let the beverages which celebrate the occasion be chosen for their peculiar and exceptional flavors. If they are good of their class, the moderate use will not shorten, but both cheer and lengthen life." But the author is, nevertheless, as unsparing in condemning the use of alcohol, as the most ardent temperance reformer could desire. Not satisfied with the researches of others, he has instituted a series of experiments himself, for the purpose of determining the true place of alcohol in dietetic economy, and has furnished the results of these experiments in tabulated form.

It would be well if the opinions expressed in the chapters upon Infant Diet, could be deeply impressed upon the minds of the rising generation of medical men—the rest are unimpressible—that the annual holocaust of infant life, offered upon the shrine of physiological ignorance, might be spared; if they could be convinced, with Dr. Chambers, that "Laputa never devised anything more preposterous" than "Liebig's food for infants," and that "it is only when the coming teeth are on their road to the front that the parotid glands secrete sufficient saliva to digest farinaceous food."

It is scarcely possible, within the limits of a brief review such as this, to do full justice to this scientific, practical, and scholarly little work. The simply practical man will find in it safe and simple rules for his guidance; the scientific man will find the demonstration of the principles upon which these rules are based; and the scholar will be able to gratify his literary and æsthetic taste in the elegant style in which they are presented. The book deserves to be read by all. H.

BOOKS RECEIVED.

TRANSACTIONS OF THE NEW YORK ODONTOLOGICAL SOCIETY—Special meeting, Dec. 14, 15, 16, 1874.

PAMPHLETS RECEIVED.

ANNUAL ADDRESS before the Society of the Alumni of the Medical Department of the University of Pennsylvania. By *Cornelius G. Comegys, M.D.*, Lecturer on Clinical Medicine in the Cincinnati Hospital, Ohio; Late Professor of the Institutes of Medicine and Clinical Medicine in the Medical College of Ohio. With the Proceedings of the Alumni Meeting of 1875.

INJECTIONS OF TINCTURE OF IODINE into the Cavity of the Uterus in Hæmorrhage, and after Delivery. By *James Trask, M.D.*, late Prof. of Obstetrics in the Long Island College Hospital. New York: William Wood & Co. 1875.

TRANSACTIONS of the Ninth Annual Meeting of the Medical Association of the State of Missouri, April 20th and 21st, 1875.

THE ALBANY MEDICAL COLLEGE—The Medical Department of Union University, Annual Catalogue and Announcement. 45th Session. 1875.

A CLINICAL CONTRIBUTION TO THE TREATMENT OF TUBAL PREGNANCY. By *T. Gaillard Thomas, M.D.* New York: D. Appleton & Co. 1875.

SYNOPSIS—TREATMENT OF UTERINE DISPLACEMENTS. By *Prof. Henry F. Campbell*, of Augusta, Georgia.

MIAMI MEDICAL COLLEGE OF CINCINNATI—Sixteenth Annual Announcement. Session 1875 and 1876.

ON THE USE OF HOT WATER IN SURGERY. By *Frank H. Hamilton*, Surgeon to Bellevue Hospital, New York. G. P. Putnam's Sons. 1875.

THE METROPOLITAN THROAT HOSPITAL, REPORT OF. *Clinton Wagner, M.D.*, Medical Supt.

THE PHYSIOLOGICAL ACTION OF THEBAINE. By *J. Ott, M.D.*, of Easton, Pa.

THE PHYSICAL PROPERTIES OF DENTAL AMALGAMS. By the late *Thomas Hitchcock, M.D., D.M.D.*

ON SPASMODIC URETHRAL STRICTURE. By *F. N. Otis, M.D.*, Clinical Professor of Genito-Urinary Diseases at the College of Physicians and Surgeons, New York. Reprinted from the Archives of Dermatology, Vol. I, No. 3. 1875.

CLINICAL STUDIES WITH THE NON-NAUSEATING USE OF IPECACUANHA, Chiefly in Intermittents. Reprinted from Atlanta Medical and Surgical Journal. 1875.

RELATIONS OF OPHTHALMOLOGY TO PRACTICAL MEDICINE. An Introductory to the Summer Course of Lectures of the Jefferson Medical College, Delivered March 29, 1875, by *William Thompson, M.D.*, Lecturer on Ophthalmic and Aural Surgery.

PATHOLOGY AND ETIOLOGY OF PULMONARY PHTHISIS, in relation to its Prevention and Early Arrest. By *E. Darwin Hudson, Jr., A.B., M.D.* New York: D. Appleton & Co. 1875.

CERTAIN NERVOUS AFFECTIONS OF THE THROAT. By *Clinton Wagner, M.D.*, Physician to the Metropolitan Throat Hospital; late Clinical Assistant, Hospital for Diseases of the Throat, London; Member of the N. Y. Laryngological Society; of the N. Y. County Medical Society; of the N. Y. Neurological Society, etc., etc. Read before the N. Y. Neurological Society, Sept. 7, 1874. Reprinted from the Psychological and Medico-Legal Journal for October, 1874. New York: F. Christern & Co., 77 University Place.

NOTES ON THE DIAGNOSIS OF BLOOD STAINS. By *Joseph G. Richardson, M.D.*, Microscopist to the Pennsylvania Hospital.

IRIDOTOMY, and its Applicability to Certain Defects of the Eye. By *W. B. Calhoun, M.D.*, Professor of Diseases of the Eye and Ear in the Atlanta Medical College. 1875.

THE DOCTRINE OF THE CORRELATION AND CONSERVATION OF FORCES, and its Bearing upon Theism. By *G. M. Duzan, M.D.*, Zionsville, Indiana. Reprinted from the Indiana Journal of Medicine.

THE EXTENSION WINDLASS, presented to the American Medical Association, May, 1875. By *Charles Dennison, M.D.*, Denver, Colorado. Reprinted from the N. Y. Medical Journal, May, 1875. New York: D. Appleton & Co.

FIFTY-FIFTH ANNUAL CATALOGUE AND ANNOUNCEMENT OF THE MEDICAL COLLEGE OF OHIO. Session 1875–6. Cincinnati.

ANNUAL CIRCULAR 1875–6. ANNUAL CATALOGUE 1874–5. Bellevue Hospital Medical College.

JOURNALS RECEIVED.

The Atlanta Medical and Surgical Journal—Vol. xiii, Nos. 3, 4.
The American Medical Weekly—Vol. ii, Nos. 21, 23, 24, 25, 26, and Vol. iii, Nos. 1, 2.
The American Practitioner—Vol. xl, Nos. 66, 67.
L'Anatomie et de la Physiologie Journal de. Ch. Robin—No. 3, Mai et Juin, Paris, 1875.
The American Journal of the Medical Sciences—No. cxxxix, July, 1875.
The Buffalo Medical and Surgical Journal—Vol. xiv, Nos. 9, 10.
The Boston Journal of Chemistry—Vol. ix, No. 12, Vol. x, No. 1.
The Canada Medical and Surgical Journal—Vol. iii, No. 12, Vol. iv, No. 1.
The Clinic—Vol. viii, Nos. 22, 23, 24, 25, 26, Vol. ix, Nos. 1, 2.
The Cincinnati Lancet and Observer—Vol. xviii, Nos. 6, 7.
The Detroit Review of Medicine and Pharmacy—Vol. x, No. 6.
The Dental Cosmos—Vol. xvii, Nos. 6, 7.
The Druggists' Circular, Vol. xix, No. 7.
The Eclectic Medical Journal—Vol. xxxv, No. 7.
The Indiana Journal of Medicine—Vol. vi, Nos. 2, 3.
The Kansas City Medical Journal—Vol. v, No. 3.
The London Lancet, June, 1875.
The Laboratory, Boston—Vol. i, No. 2.
The Medical News and Library—Vol. xxxiii, Nos. 389, 391.
The Monthly Abstract of Medical Sciences—Vol. ii, Nos. 5, 6, 7.
The Medical Record, New York—Vol. x, Nos. 22, 23, 24, 25, 26, 27, 28.
The Medical and Surgical Reporter, Philadelphia—Vol. xxxii, Nos. 22, 23, 24, 25, 26, Vol. xxxiii, Nos. 1, 2

The Medical Times, Philad.—Vol. v, Nos. 187, 188, 189, 190, 191, 192, 193.
The Melbourne Medical Record—Vol. iv, Nos, 7, 8, 9, 10.
The Medical Herald—Vol. viii, No. 2.
The Medical Examiner, Chicago—Vol. xvi, Nos. 11, 12, 13.
The Medical Press and Circular, London—Vol. xviii, No. 1896.
The Medical Times and Gazette, May 29, 1875.
The Medical Register and Advertiser—Vol. i, No. 2.
The Nashville Journal of Medicine and Surgery— Vol. xxxvi, No. 6, Vol. xxxvii, No. 1.
The New York Medical Journal—Vol. xxi, No. 6, Vol. xxii, No. 1.
The New Orleans Medical and Surgical Journal—Vol. iii, No. 1.
The Pharmacist—Vol. viii, No. 7.
The Pacific Medical and Surgical Journal—Vol. xviii, Nos. 1, 2.
The Practitioner, London—Nos. lxxxiii and lxxxiv.
The Printer Artisan—Vol. iii, No. 1.
Le Progres Medicale, Paris, 3c Annee—Nos. 16, 17, 18, 19, 20.
The Public Health Magazine, Montreal—Vol i, No. 1.
The Richmond and Louisville Medical and Surgical Journal—Vol xix, Nos. 5, 6.
The St. Louis Medical and Surgical Journal—Vol. xii, Nos. 6, 7.
The Southern Medical Record—Vol. v, No. 4.
The St. Louis Medical Record—Vol. ii, No. 3.
The Sanitary Journal—Vol. 1, No. 7
The St. Louis Clinical Record—Vol. ii, No. 4.
The Technologist—Vol. vi, No. 6.
The Texas Medical Journal—Vol iii, No. 2.
The Virginia Medical Monthly—Vol. ii, Nos. 3, 4.

